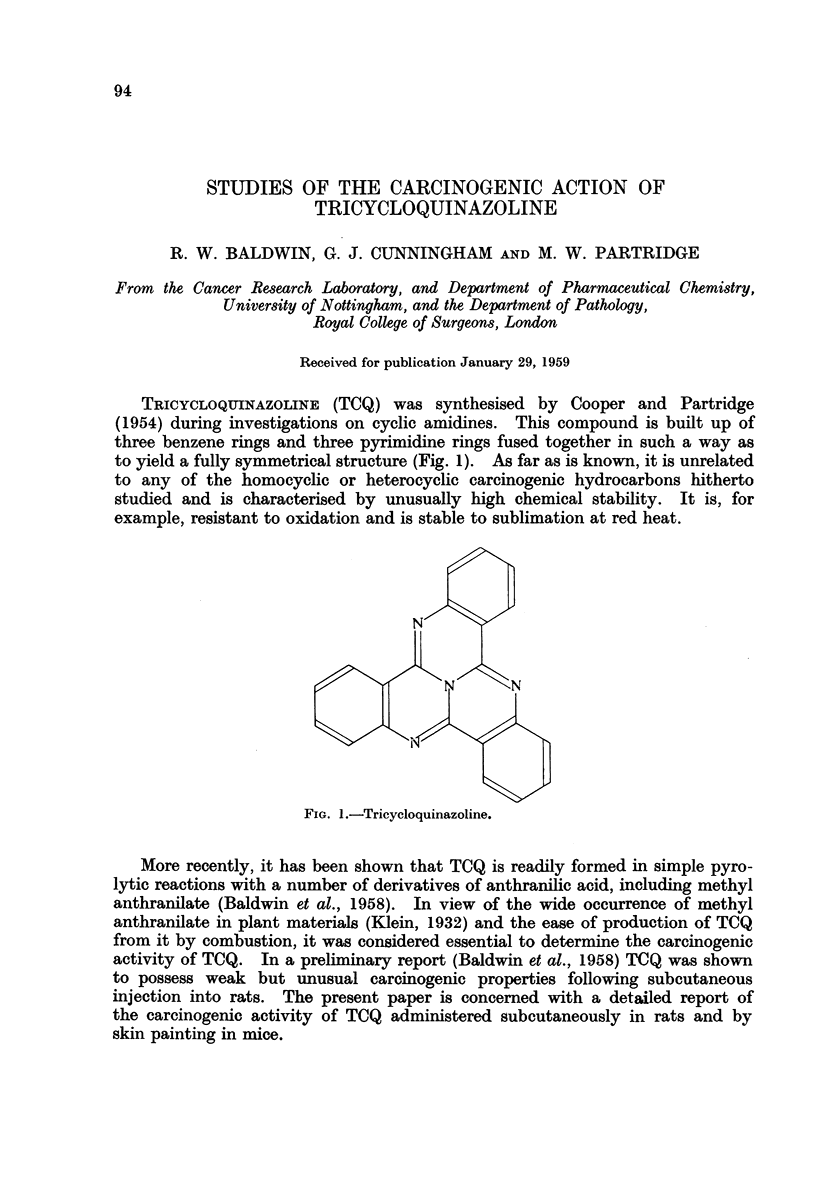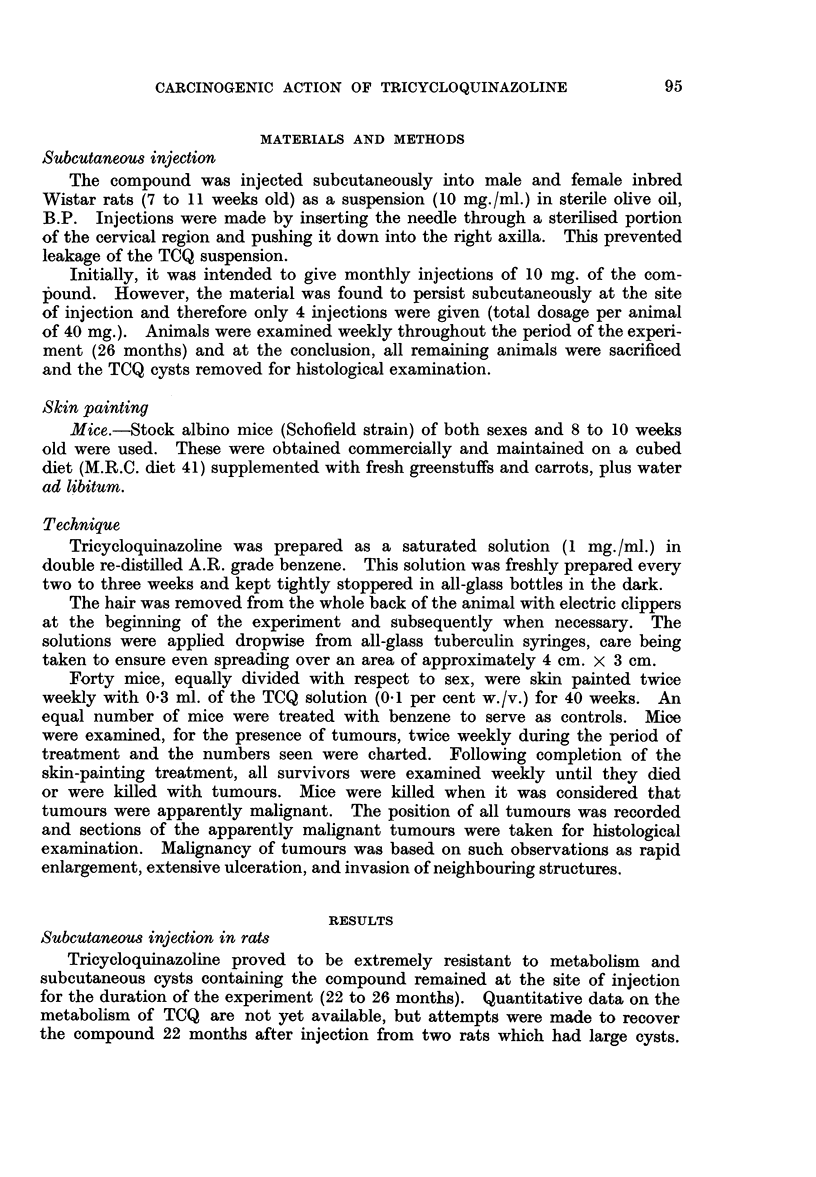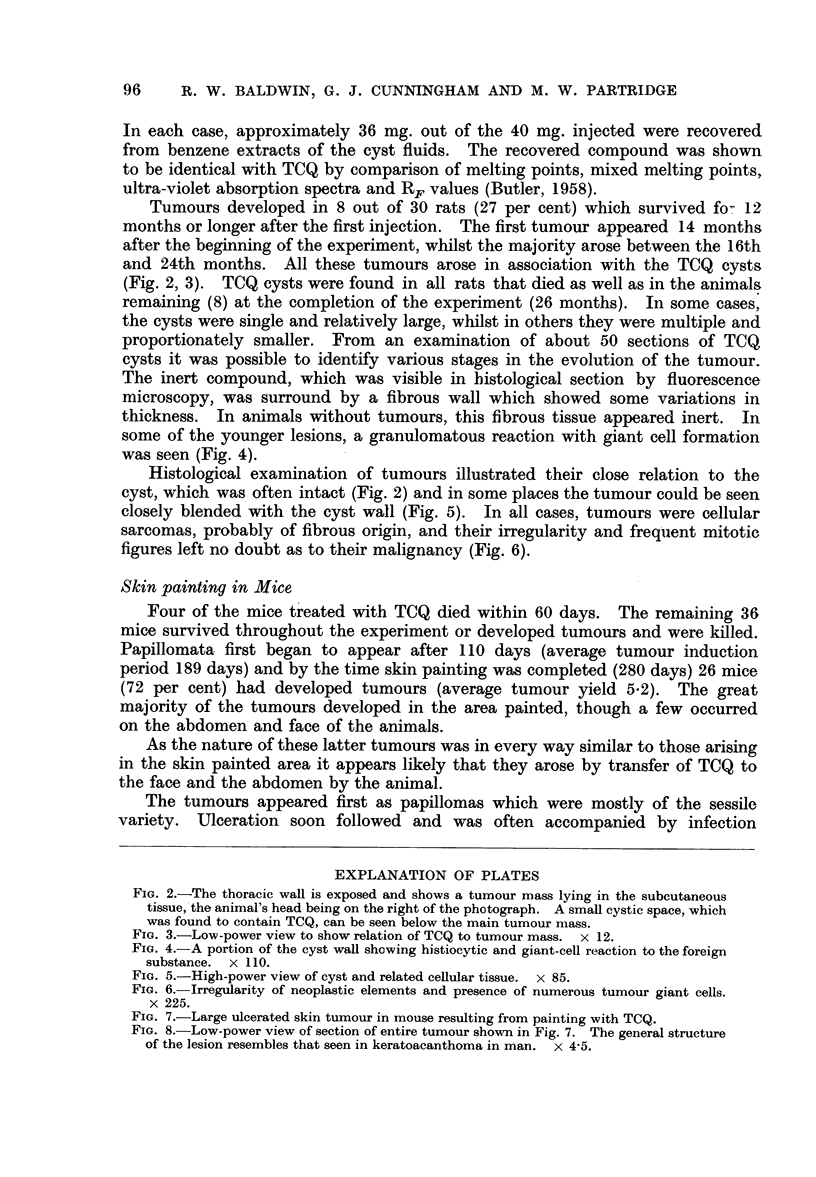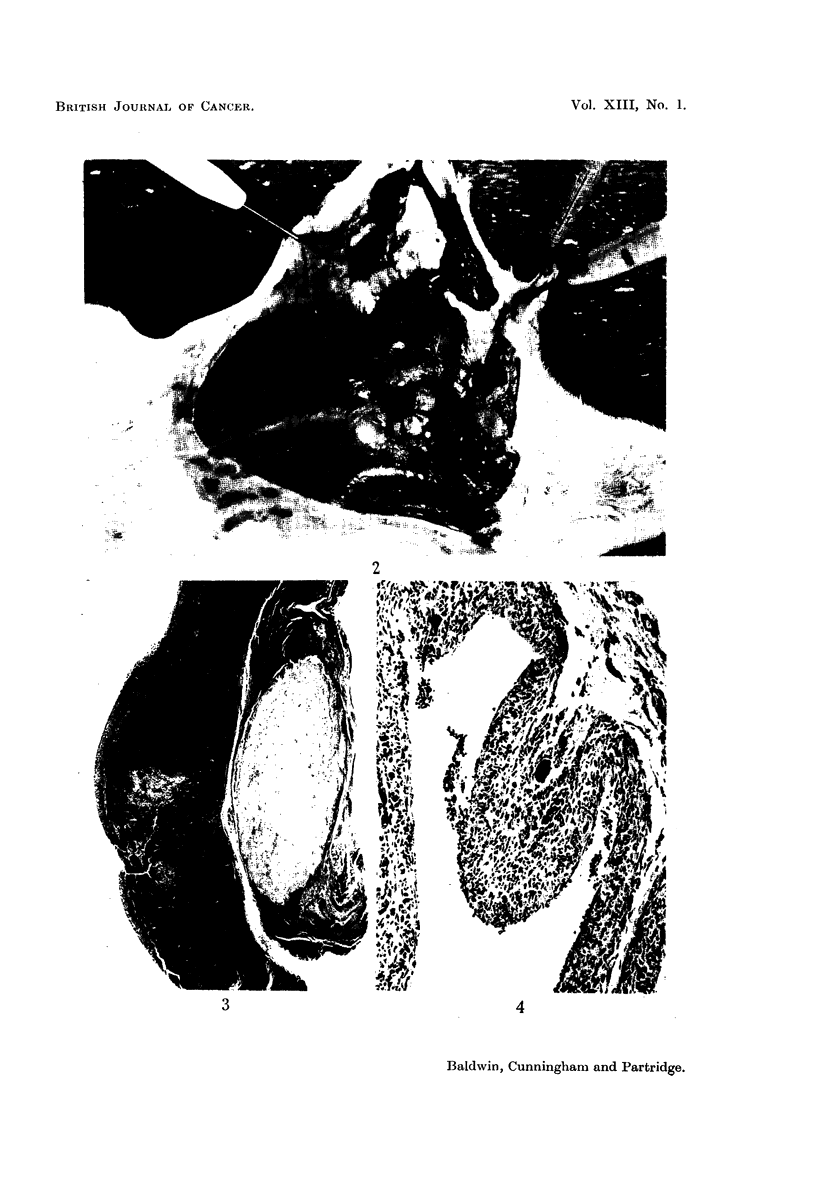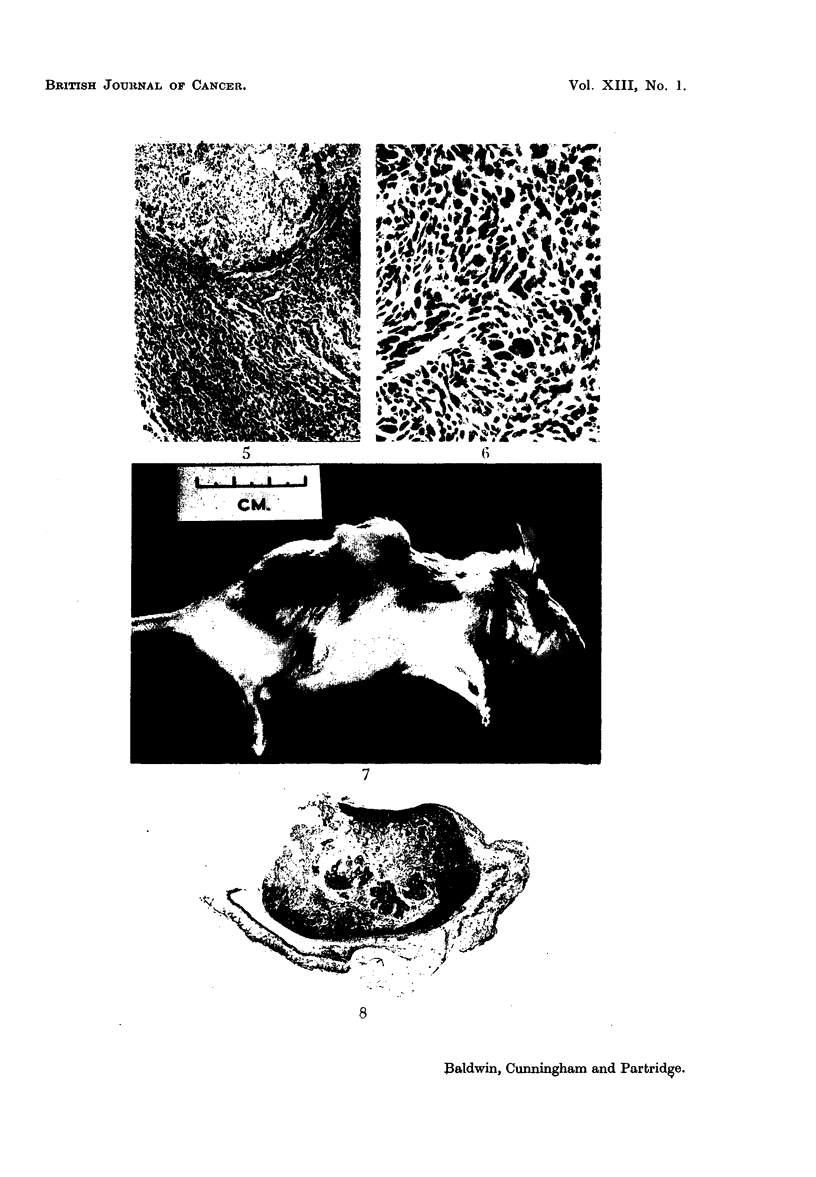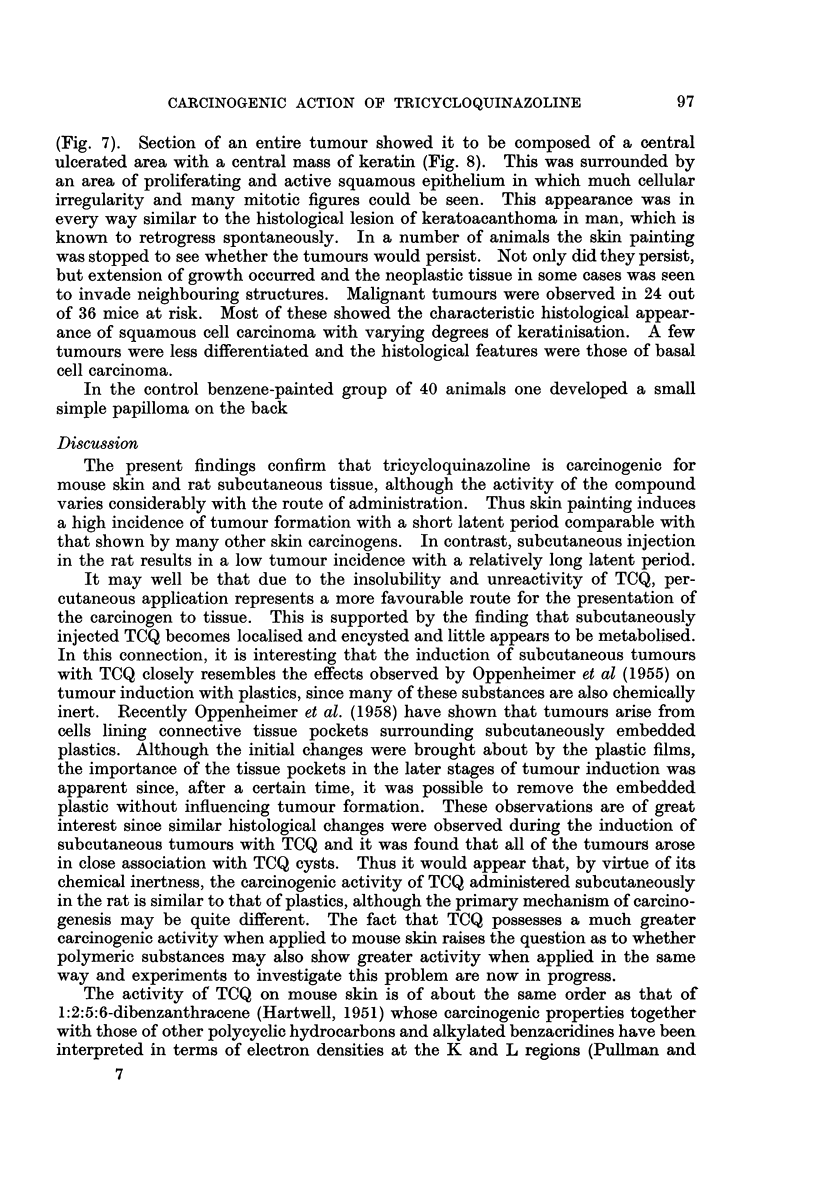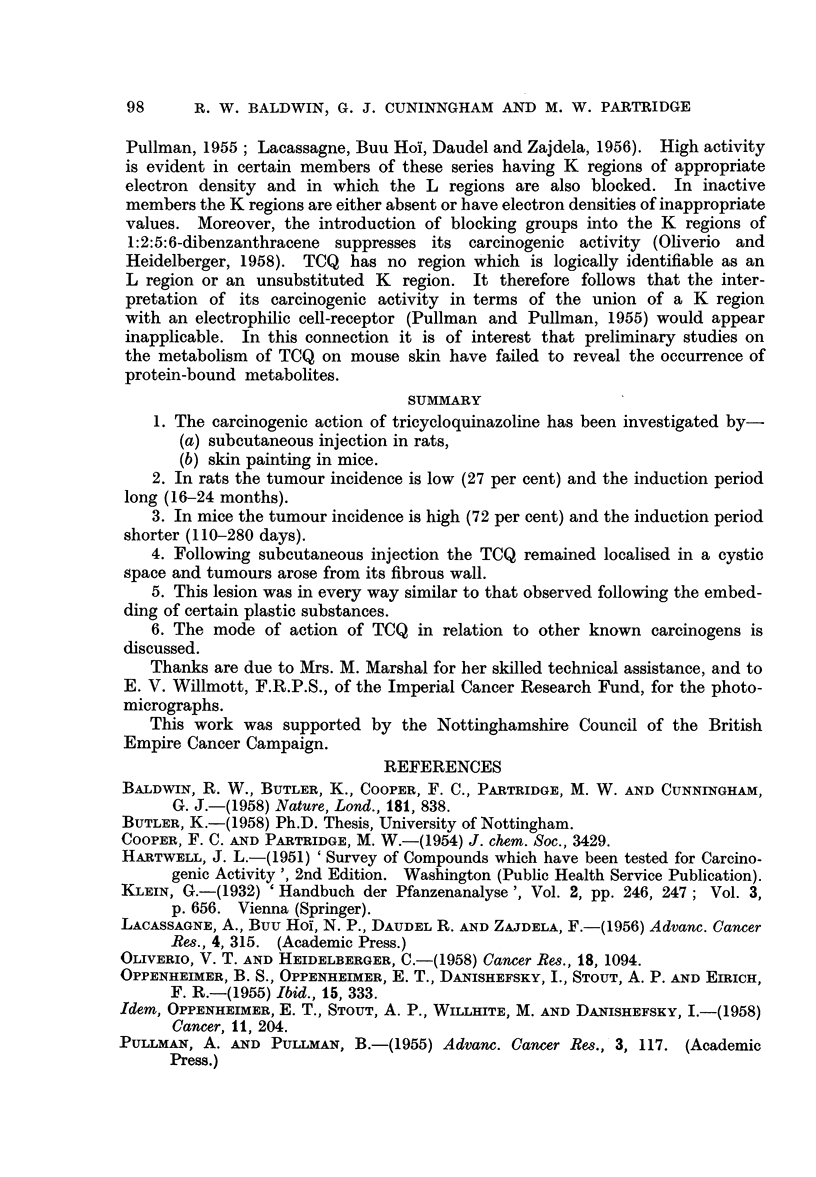# Studies of the Carcinogenic Action of Tricycloquinazoline

**DOI:** 10.1038/bjc.1959.13

**Published:** 1959-03

**Authors:** R. W. Baldwin, G. J. Cunningham, M. W. Partridge

## Abstract

**Images:**


### STUDIES OF THE CARCINOGENIC ACTION OF TRICYCLOQUINAZOLINE

R. W. BALDWIN, G. J. CUNNINGHAM AND M. W. PARTRIDGE

*From the Cancer Research Laboratory, and Department of Pharmaceutical Chemistry, University of Nottingham, and the Department of Pathology, Royal College of Surgeons, London*

Received for publication January 29, 1959

TRICYCLOQUINAZOLINE (TCQ) was synthesised by Cooper and Partridge (1954) during investigations on cyclic amidines. This compound is built up of three benzene rings and three pyrimidine rings fused together in such a way as to yield a fully symmetrical structure (Fig. 1). As far as is known, it is unrelated to any of the homocyclic or heterocyclic carcinogenic hydrocarbons hitherto studied and is characterised by unusually high chemical stability. It is, for example, resistant to oxidation and is stable to sublimation at red heat.

FIG. 1.—Tricycloquinazoline.

More recently, it has been shown that TCQ is readily formed in simple pyrolytic reactions with a number of derivatives of anthranilic acid, including methyl anthranilate (Baldwin *et al.*, 1958). In view of the wide occurrence of methyl anthranilate in plant materials (Klein, 1932) and the ease of production of TCQ from it by combustion, it was considered essential to determine the carcinogenic activity of TCQ. In a preliminary report (Baldwin *et al.*, 1958) TCQ was shown to possess weak but unusual carcinogenic properties following subcutaneous injection into rats. The present paper is concerned with a detailed report of the carcinogenic activity of TCQ administered subcutaneously in rats and by skin painting in mice.

### MATERIALS AND METHODS

### *Subcutaneous injection*

The compound was injected subcutaneously into male and female inbred Wistar rats (7 to 11 weeks old) as a suspension (10 mg./ml.) in sterile olive oil, B.P. Injections were made by inserting the needle through a sterilised portion of the cervical region and pushing it down into the right axilla. This prevented leakage of the TCQ suspension.

Initially, it was intended to give monthly injections of 10 mg. of the compound. However, the material was found to persist subcutaneously at the site of injection and therefore only 4 injections were given (total dosage per animal of 40 mg.). Animals were examined weekly throughout the period of the experiment (26 months) and at the conclusion, all remaining animals were sacrificed and the TCQ cysts removed for histological examination.

### *Skin painting*

*Mice.*—Stock albino mice (Schofield strain) of both sexes and 8 to 10 weeks old were used. These were obtained commercially and maintained on a cubed diet (M.R.C. diet 41) supplemented with fresh greenstuffs and carrots, plus water *ad libitum.*

### *Technique*

Tricycloquinazoline was prepared as a saturated solution (1 mg./ml.) in double re-distilled A.R. grade benzene. This solution was freshly prepared every two to three weeks and kept tightly stoppered in all-glass bottles in the dark.

The hair was removed from the whole back of the animal with electric clippers at the beginning of the experiment and subsequently when necessary. The solutions were applied dropwise from all-glass tuberculin syringes, care being taken to ensure even spreading over an area of approximately 4 cm. × 3 cm.

Forty mice, equally divided with respect to sex, were skin painted twice weekly with 0·3 ml. of the TCQ solution (0·1 per cent w./v.) for 40 weeks. An equal number of mice were treated with benzene to serve as controls. Mice were examined, for the presence of tumours, twice weekly during the period of treatment and the numbers seen were charted. Following completion of the skin-painting treatment, all survivors were examined weekly until they died or were killed with tumours. Mice were killed when it was considered that tumours were apparently malignant. The position of all tumours was recorded and sections of the apparently malignant tumours were taken for histological examination. Malignancy of tumours was based on such observations as rapid enlargement, extensive ulceration, and invasion of neighbouring structures.

### RESULTS

### *Subcutaneous injection in rats*

Tricycloquinazoline proved to be extremely resistant to metabolism and subcutaneous cysts containing the compound remained at the site of injection for the duration of the experiment (22 to 26 months). Quantitative data on the metabolism of TCQ are not yet available, but attempts were made to recover the compound 22 months after injection from two rats which had large cysts.

In each case, approximately 36 mg. out of the 40 mg. injected were recovered from benzene extracts of the cyst fluids. The recovered compound was shown to be identical with TCQ by comparison of melting points, mixed melting points, ultra-violet absorption spectra and $R_F$ values (Butler, 1958).

Tumours developed in 8 out of 30 rats (27 per cent) which survived for 12 months or longer after the first injection. The first tumour appeared 14 months after the beginning of the experiment, whilst the majority arose between the 16th and 24th months. All these tumours arose in association with the TCQ cysts (Fig. 2, 3). TCQ cysts were found in all rats that died as well as in the animals remaining (8) at the completion of the experiment (26 months). In some cases, the cysts were single and relatively large, whilst in others they were multiple and proportionately smaller. From an examination of about 50 sections of TCQ cysts it was possible to identify various stages in the evolution of the tumour. The inert compound, which was visible in histological section by fluorescence microscopy, was surround by a fibrous wall which showed some variations in thickness. In animals without tumours, this fibrous tissue appeared inert. In some of the younger lesions, a granulomatous reaction with giant cell formation was seen (Fig. 4).

Histological examination of tumours illustrated their close relation to the cyst, which was often intact (Fig. 2) and in some places the tumour could be seen closely blended with the cyst wall (Fig. 5). In all cases, tumours were cellular sarcomas, probably of fibrous origin, and their irregularity and frequent mitotic figures left no doubt as to their malignancy (Fig. 6).

*Skin painting in Mice*

Four of the mice treated with TCQ died within 60 days. The remaining 36 mice survived throughout the experiment or developed tumours and were killed. Papillomata first began to appear after 110 days (average tumour induction period 189 days) and by the time skin painting was completed (280 days) 26 mice (72 per cent) had developed tumours (average tumour yield 5·2). The great majority of the tumours developed in the area painted, though a few occurred on the abdomen and face of the animals.

As the nature of these latter tumours was in every way similar to those arising in the skin painted area it appears likely that they arose by transfer of TCQ to the face and the abdomen by the animal.

The tumours appeared first as papillomas which were mostly of the sessile variety. Ulceration soon followed and was often accompanied by infection

---

EXPLANATION OF PLATES

FIG. 2.—The thoracic wall is exposed and shows a tumour mass lying in the subcutaneous tissue, the animal's head being on the right of the photograph. A small cystic space, which was found to contain TCQ, can be seen below the main tumour mass.

FIG. 3.—Low-power view to show relation of TCQ to tumour mass. × 12.

FIG. 4.—A portion of the cyst wall showing histiocytic and giant-cell reaction to the foreign substance. × 110.

FIG. 5.—High-power view of cyst and related cellular tissue. × 85.

FIG. 6.—Irregularity of neoplastic elements and presence of numerous tumour giant cells. × 225.

FIG. 7.—Large ulcerated skin tumour in mouse resulting from painting with TCQ.

FIG. 8.—Low-power view of section of entire tumour shown in Fig. 7. The general structure of the lesion resembles that seen in keratoacanthoma in man. × 4·5.

2

3

4

Baldwin, Cunningham and Partridge.

5

6

7

8

Baldwin, Cunningham and Partridge.

(Fig. 7). Section of an entire tumour showed it to be composed of a central ulcerated area with a central mass of keratin (Fig. 8). This was surrounded by an area of proliferating and active squamous epithelium in which much cellular irregularity and many mitotic figures could be seen. This appearance was in every way similar to the histological lesion of keratoacanthoma in man, which is known to retrogress spontaneously. In a number of animals the skin painting was stopped to see whether the tumours would persist. Not only did they persist, but extension of growth occurred and the neoplastic tissue in some cases was seen to invade neighbouring structures. Malignant tumours were observed in 24 out of 36 mice at risk. Most of these showed the characteristic histological appearance of squamous cell carcinoma with varying degrees of keratinisation. A few tumours were less differentiated and the histological features were those of basal cell carcinoma.

In the control benzene-painted group of 40 animals one developed a small simple papilloma on the back

*Discussion*

The present findings confirm that tricycloquinazoline is carcinogenic for mouse skin and rat subcutaneous tissue, although the activity of the compound varies considerably with the route of administration. Thus skin painting induces a high incidence of tumour formation with a short latent period comparable with that shown by many other skin carcinogens. In contrast, subcutaneous injection in the rat results in a low tumour incidence with a relatively long latent period.

It may well be that due to the insolubility and unreactivity of TCQ, percutaneous application represents a more favourable route for the presentation of the carcinogen to tissue. This is supported by the finding that subcutaneously injected TCQ becomes localised and encysted and little appears to be metabolised. In this connection, it is interesting that the induction of subcutaneous tumours with TCQ closely resembles the effects observed by Oppenheimer *et al* (1955) on tumour induction with plastics, since many of these substances are also chemically inert. Recently Oppenheimer *et al.* (1958) have shown that tumours arise from cells lining connective tissue pockets surrounding subcutaneously embedded plastics. Although the initial changes were brought about by the plastic films, the importance of the tissue pockets in the later stages of tumour induction was apparent since, after a certain time, it was possible to remove the embedded plastic without influencing tumour formation. These observations are of great interest since similar histological changes were observed during the induction of subcutaneous tumours with TCQ and it was found that all of the tumours arose in close association with TCQ cysts. Thus it would appear that, by virtue of its chemical inertness, the carcinogenic activity of TCQ administered subcutaneously in the rat is similar to that of plastics, although the primary mechanism of carcinogenesis may be quite different. The fact that TCQ possesses a much greater carcinogenic activity when applied to mouse skin raises the question as to whether polymeric substances may also show greater activity when applied in the same way and experiments to investigate this problem are now in progress.

The activity of TCQ on mouse skin is of about the same order as that of 1:2:5:6-dibenzanthracene (Hartwell, 1951) whose carcinogenic properties together with those of other polycyclic hydrocarbons and alkylated benzacridines have been interpreted in terms of electron densities at the K and L regions (Pullman and

Pullman, 1955 ; Lacassagne, Buu Hoï, Daudel and Zajdela, 1956). High activity is evident in certain members of these series having K regions of appropriate electron density and in which the L regions are also blocked. In inactive members the K regions are either absent or have electron densities of inappropriate values. Moreover, the introduction of blocking groups into the K regions of 1:2:5:6-dibenzanthracene suppresses its carcinogenic activity (Oliverio and Heidelberger, 1958). TCQ has no region which is logically identifiable as an L region or an unsubstituted K region. It therefore follows that the interpretation of its carcinogenic activity in terms of the union of a K region with an electrophilic cell-receptor (Pullman and Pullman, 1955) would appear inapplicable. In this connection it is of interest that preliminary studies on the metabolism of TCQ on mouse skin have failed to reveal the occurrence of protein-bound metabolites.

### SUMMARY

1. The carcinogenic action of tricycloquinazoline has been investigated by—
   (*a*) subcutaneous injection in rats,
   (*b*) skin painting in mice.

2. In rats the tumour incidence is low (27 per cent) and the induction period long (16–24 months).

3. In mice the tumour incidence is high (72 per cent) and the induction period shorter (110–280 days).

4. Following subcutaneous injection the TCQ remained localised in a cystic space and tumours arose from its fibrous wall.

5. This lesion was in every way similar to that observed following the embedding of certain plastic substances.

6. The mode of action of TCQ in relation to other known carcinogens is discussed.

Thanks are due to Mrs. M. Marshal for her skilled technical assistance, and to E. V. Willmott, F.R.P.S., of the Imperial Cancer Research Fund, for the photomicrographs.

This work was supported by the Nottinghamshire Council of the British Empire Cancer Campaign.

### REFERENCES

BALDWIN, R. W., BUTLER, K., COOPER, F. C., PARTRIDGE, M. W. AND CUNNINGHAM, G. J.—(1958) *Nature, Lond.*, **181**, 838.

BUTLER, K.—(1958) Ph.D. Thesis, University of Nottingham.

COOPER, F. C. AND PARTRIDGE, M. W.—(1954) *J. chem. Soc.*, 3429.

HARTWELL, J. L.—(1951) ‘ Survey of Compounds which have been tested for Carcinogenic Activity ’, 2nd Edition. Washington (Public Health Service Publication).

KLEIN, G.—(1932) ‘ Handbuch der Pfanzenanalyse ’, Vol. **2**, pp. 246, 247 ; Vol. **3**, p. 656. Vienna (Springer).

LACASSAGNE, A., BUU HOÏ, N. P., DAUDEL R. AND ZAJDELA, F.—(1956) *Advanc. Cancer Res.*, **4**, 315. (Academic Press.)

OLIVERIO, V. T. AND HEIDELBERGER, C.—(1958) *Cancer Res.*, **18**, 1094.

OPPENHEIMER, B. S., OPPENHEIMER, E. T., DANISHEFSKY, I., STOUT, A. P. AND EIRICH, F. R.—(1955) *Ibid.*, **15**, 333.

*Idem*, OPPENHEIMER, E. T., STOUT, A. P., WILLHITE, M. AND DANISHEFSKY, I.—(1958) *Cancer*, **11**, 204.

PULLMAN, A. AND PULLMAN, B.—(1955) *Advanc. Cancer Res.*, **3**, 117. (Academic Press.)